# Perceived Environment in Psychiatric Rehabilitation: Implications for Recovery

**DOI:** 10.1192/j.eurpsy.2026.11700

**Published:** 2026-07-31

**Authors:** A. Vacca, M. V. Mino’, F. Franza, A. Litta

**Affiliations:** 1 Mental Health Department, Asl Taranto, Taranto; 2 Psychiatric Rehabilitation Center “Don Tonino Bello”- Assoc. M.I.T.A.G., Brindisi; 3 ”Psychiatric Studies Center" (Cen.Stu.Psi.), Provaglio d’ Iseo (BS); 4 Department of Precision and Regenerative Medicine and Ionian Area (DiMePRe-J), University of Bari, Bari, Italy

## Abstract

**Introduction:**

The physical environment of psychiatric residential facilities can act as either a facilitator or a barrier in recovery pathways. Recovery, as a paradigm, emphasizes autonomy, hope, and empowerment beyond symptom reduction. Understanding how patients perceive their environment may provide innovative strategies to strengthen recovery-oriented rehabilitation.

**Objectives:**

To explore the role of subjective environmental perception in psychiatric rehabilitation and to propose practical tools—such as Post-Occupancy Evaluation (POE)—to integrate patient experience into recovery-oriented care

**Methods:**

A narrative review of the international literature on recovery and environmental factors was conducted, including studies applying POE in healthcare contexts (Davidson et al. 2005; Topor et al. 2011; Ferrante & Villani 2020). The analysis focused on patient involvement in environmental assessment and on the development of user-centred evaluation tools.

**Results:**

Evidence highlights that factors such as privacy, choice, natural light, aesthetic comfort, and perceived safety enhance Recovery. Direct involvement of patients and staff through POE (Post-Occupancy Evaluation) methodologies—such as focus groups, guided walk-throughs, and user-friendly questionnaires—emerges as a promising strategy to empower individuals and to improve the quality of rehabilitation environments. The proposal of a structured yet flexible questionnaire targeting accessibility, comfort, autonomy, personalization, social interaction, and safety may foster patient awareness and facilitate dialogue with professionals. Integrating subjective perception into rehabilitation pathways not only improves the immediate therapeutic environment but also equips patients with environmental self-regulation skills, supporting stability and quality of life after discharge.

**Conclusions:**

The perceived quality of the therapeutic environment is a strategic factor for the success of recovery in psychiatric rehabilitation. Including POE tools and participatory assessment processes in individualized care plans represents an innovative and sustainable approach to strengthen patient empowerment and promote long-term recovery.

**Disclosure of Interest:**

None Declared

